# Chrysin attenuates intervertebral disk degeneration via dual inhibition of matrix metalloproteinases and senescence: integrated network pharmacology, molecular docking, and experimental validation

**DOI:** 10.3389/fmed.2025.1593317

**Published:** 2025-05-12

**Authors:** Zeyu Pang, Junxian Hu, Chen Zhao, Xiaoxiao Li, Yibo Zhu, Xiangwei Li, Yiyang Wang, Qiang Zhou, Pei Li

**Affiliations:** ^1^Department of Orthopedics, The Third Affiliated Hospital of Chongqing Medical University, Chongqing, China; ^2^Tissue Repairing and Biotechnology Research Center, The Third Affiliated Hospital of Chongqing Medical University, Chongqing, China

**Keywords:** Chrysin, network pharmacology, intervertebral disk degeneration, aging and degeneration, molecular docking, molecular dynamics simulations

## Abstract

Intervertebral disk degeneration (DDD) caused by nucleus pulposus cell (NPCs) senescence, oxidative stress, and extracellular matrix (ECM) degradation is one of the leading causes of chronic low back pain, yet effective treatments remain elusive. This study investigated the potential of chrysin, a natural flavonoid with antioxidant and anti-inflammatory properties, to alleviate NPCs aging and ECM dysregulation. Through network pharmacology, researchers identified 89 overlapping targets between chrysin and DDD, including MMP2, MMP9, and TGFB1. Enrichment analyses revealed key pathways in cancer, such as JAK-STAT signaling, efflux cells, and central carbon metabolism. Molecular docking showed that chrysin has a strong binding affinity for MMP2 (–8.4 kcal/mol) and MMP9 (–8.2 kcal/mol), key enzymes for ECM degradation. Molecular dynamics simulations demonstrated that the Chrysin-MMP-9 and Chrysin-MMP-2 complexes exhibited favorable dynamic properties. Experimental validation in H2O2-induced senescent NPCs confirmed the protective effects of chrysin: pretreatment with chrysin (1 μM) significantly reduced senescence-associated β-galactosidase activity and inhibited MMP2/9 mRNA expression while restoring collagen II and aggrecan levels. In addition, Chrysin attenuated oxidative stress-mediated ECM damage, which was consistent with network predictions. These findings highlight the dual ability of Chrysin to inhibit MMP activity and combat aging, making it a promising multi-targeted therapeutic candidate for the treatment of DDD. This study combines bioinformatics with experimental modeling to mechanistically reveal the anti-aging mechanism of Chrysin.

## 1 Introduction

Low back pain (LBP) stands as the primary contributor to disability worldwide, placing a significant strain on both socioeconomic structures and healthcare systems (1). Studies reveal that a staggering 84% of adults experience LBP at some point in their lives, with roughly 23% facing ongoing disruptions to their work and personal lives due to its persistent effects (2). Intervertebral disc degeneration (DDD), characterized by extracellular matrix (ECM) catabolism, oxidative stress, and nucleus pulposus (NP) cell senescence, is a leading cause of chronic low back pain (3). Under pathological conditions, excessive reactive oxygen species (ROS) drive NP cell senescence and upregulate matrix metalloproteinases (MMPs), which directly degrade type II collagen and aggrecan – the core structural components of disk ECM (4, 5). Unfortunately, current approaches to managing LBP remain largely confined to medications or surgical interventions aimed at symptom relief, with no definitive treatment available to address DDD itself. This gap stems from the intricate and multifaceted nature of DDD’s underlying causes (6). Consequently, delving deeper into the molecular mechanisms of DDD holds immense clinical importance and could pave the way for more effective solutions.

Aging ranks among the primary factors influencing DDD (7). As the years go by, the quality, levels of anabolism, and biomechanical properties of the extracellular matrix (ECM) take a nosedive, while catabolic processes and senescent cells (8, 9) experience an uptick. In the context of DDD, inflammatory factors secreted by senescent nucleus pulposus cells (NPCss) trigger the expression of matrix metalloproteinases (MMPs), thereby hastening the degradation of the ECM (10). Moreover, the breakdown of the ECM results in a decline in the water content within the NP. This not only impacts the height of the intervertebral disk but also ramps up the shear force acting on the NPCss. Ultimately, this leads to the transformation of collagen type II (Col - II) into collagen type I (Col - I), accelerating NPCs senescence and exacerbating DDD (11, 12). Consequently, putting the brakes on NPCs senescence could well be an effective strategy for treating DDD.

Cellular senescence represents an unyielding halt in the cell cycle. It’s regarded as one of the key culprits behind numerous chronic degenerative maladies, DDD among them (13). The tell - tale signs of cell senescence are the uptick in senescence - associated β - galactosidase (SA - β - Gal) positive cells and the heightened expression of cyclin - dependent kinase (CDK) inhibitors like p16INK4a (14). The inhibition of the CDK - cyclin complex is the primary driver of cell proliferation arrest (15). Moreover, senescent cells can secrete the senescence - associated secretory phenotype (SASP), which has an impact on the cellular microenvironment and speeds up the senescence of neighboring cells. SASP chiefly consists of MMPs and inflammatory factors such as high mobility group protein b1 (HMGB1) (16). There are multiple factors contributing to cellular senescence, oxidative stress being one of them (17). Supanji et al. demonstrated that oxidative stress can activate p16INK4a via MAPK, which then triggers cell senescence (18). Nevertheless, the connection between oxidative stress and cellular senescence still needs to be delved into more deeply.

Chrysin, also known as 5, 7-dihydroxyflavone, is a flavonoid extracted from the Bignoniaceae plant oryx and is relatively high in propolis (19, 20). It has a wide range of pharmacological and physiological activities such as anti-oxidation, anti-tumor, anti-hypertension, anti-diabetes, anti-bacteria, and anti-allergy (21, 22). In view of its wide distribution and relatively low toxicity, chrysin has been considered a promising therapeutic agent for various diseases (19, 23, 24). Recent advances in flavonoid-based therapeutics have highlighted their potential in combating DDD. Studies demonstrate that hesperidin attenuates ferroptosis in NPCs through the Nrf2/NF-κB axis (25), while Rhizoma drynariae total flavonoids mitigate matrix degradation via MAPK pathway inhibition in cervical disk models (26). Similarly, grape seed extract and naringin exhibit anti-apoptotic and anti-inflammatory effects in both rabbit and human NPCs (27, 28). These findings collectively underscore flavonoids’ capacity to target key DDD pathways – oxidative stress, inflammation, and ECM dysregulation. However, studies on the roles of Chrysin in anti-aging and anti-degeneration in NPCs are lacking.

The swift advancement of bioinformatics has paved the way for network pharmacology, grounded in extensive databases, to become a formidable instrument for meticulously elucidating the mechanisms through which complex drug systems operate, spanning from the molecular to the pathway level (29). Network pharmacology is particularly effective for the analysis of multi-targeted agents, as it harmonizes various interdisciplinary technologies, including systems biology, poly-pharmacology, molecular network data, bioinformatics, and computational simulation. Numerous studies have leveraged network pharmacology methods to uncover the intricacies of how drugs affect diseases. Concurrently, this approach has emerged as a promising strategy to expedite the drug research and development process (30). Consequently, integrating network pharmacology and bioinformatics in traditional Chinese medicine research could prove fruitful in pinpointing therapeutic targets for DDD. In this study, network pharmacology, molecular docking and molecular dynamics simulations were merged to uncover the latent pharmacological effect of Chrysin in retarding aging and degeneration in NPCs by suppressing the expression of MMPs. Moreover, the mechanism was verified by employing an H_2_O_2_ - induced model of aging and degeneration in NPCs.

## 2 Materials and methods

### 2.1 Nucleus pulposus cell culture

Nucleus pulposus (NP) cells were isolated from 8 weeks-old male Sprague-Dawley rats (*n* = 6). Under aseptic conditions, caudal intervertebral disks were surgically exposed by careful removal of paraspinal musculature. NP tissues were excised via disk transection, enzymatically digested with 0.2% collagenase II in DMEM/F-12 at 37°C for 30 min with gentle agitation, and mechanically dissociated using microsurgical scissors. Following centrifugation (300 × *g*, 5 min), the cellular pellet was resuspended in complete growth medium (DMEM/F-12 supplemented with 15% FBS, 1% penicillin-streptomycin). Primary cultures were maintained in a 5% CO2 humidified incubator at 37°C. Cells at 90% confluence were subcultured, with passages 2–3 (P2–P3) selected for experimental use. All procedures were approved by the Institutional Animal Care and Use Committee (IACUC) of Chongqing Medical University (CQMU).

### 2.2 Cell viability analysis

In accordance with the manufacturer’s protocol (Dojindo, Rockville, MD, United States), the Cell Counting Kit-8 (CCK-8) assay was performed. Nucleus pulposus cell (NPC) suspensions were seeded into 96-well plates at a density of 500 cells/well. After cellular adhesion, cells were treated with 75 μM H_2_O_2_ [a concentration validated in recent intervertebral disk degeneration-senescence models (31)] for 24 h, followed by medium replacement. Add different concentrations of Chrysin (1, 10, 100 μM), chrysin (Cas No, 480-40-0, Co, United States) were provided from Sigma-Aldrich. Chrysin was dissolved in 0.5% methylcellulose (32). Cells were incubated for 1, 3, or 5 days. Post-treatment, wells were gently rinsed with phosphate-buffered saline (PBS) and supplemented with 100 μL DMEM/F-12 medium containing 10 μL CCK-8 reagent. Following a 2–4 h incubation at 37°C, absorbance was quantified at 450 nm using a microplate spectrophotometer.

### 2.3 SA-β-Gal staining

Following treatment, cells were fixed with 4% paraformaldehyde (SA-β-Gal Staining Fixative, Beyotime) at room temperature (25°C) for 15 min. Subsequently, cells were washed thrice with PBS (pH 7.4) and incubated with β-galactosidase staining solution (Beyotime) according to the manufacturer’s protocol. The staining reaction proceeded in a dry incubator at 37°C (non-CO2 atmosphere) for 24 h. Cellular senescence was quantified by counting SA-β-gal-positive cells under an inverted phase-contrast microscope with five random fields analyzed per well.

### 2.4 RT-PCR

Total RNA was extracted from nucleus pulposus (NP) cells using TRIzol™ Reagent (Invitrogen, United States) according to the manufacturer’s protocol. RNA concentration and purity were quantified spectrophotometrically (A260/A280 ratio = 1.8), followed by reverse transcription into cDNA using the Transcriptor First Strand cDNA Synthesis Kit (Roche, Switzerland). Quantitative reverse transcription polymerase chain reaction (qRT-PCR) was performed on a LightCycler^®^ 480 System (Roche) with SYBR Green I Master Mix. Each 20 μL reaction contained 100 ng cDNA, 0.5 μM gene-specific primers (Table 1), and 1x Master Mix, under the following cycling parameters: initial denaturation at 95°C for 10 min, followed by 40 cycles of 95°C for 15 s and 60°C for 1 min. GAPDH mRNA expression served as the normalization control, with relative gene expression calculated via the 2^–ΔΔCt^ method.

**TABLE 1:** Primers of target genes.

Gene	Forward (5’–3’)	Reverse (5’–3’)
GAPDH	GCAAGTTCAACGG CACAG	CGCCAGTAGAC TCCACGAC
MMP9	CTACACGGAGCATGG CAACGG	TGGTGCAGGCAGAGT AGGAGTG
ACAN	GCGTGTGCCAGAAG ACCAGAAG	ATGTCCTCTTCACCA CCCACTCC
COL II	GGAGCAGCAAGAG CAAGGAGAAG	GGAGCCCTCAGTG GACAGTAGAC
MMP2	ATGGCATTGCTCAGATCCGT	AGCCTTCTCTTCCT GTGGGG

### 2.6 Acquisition of Chrysin targets

The search for chrysin’s targets was conducted using three key databases, all of which involved the keyword “Chrysin.” The first resource utilized was the TCMSP database, which primarily provides target information derived from the HIT database, the SysDT model, and various experimental validations. Next, the SuperPred database was explored to identify possible therapeutic drug targets. Additionally, the SMILE notation for Chrysin was retrieved from the PubChem database, followed by gathering the corresponding drug target information from the Swiss Target Prediction database. Ultimately, the data collected from these three sources was transformed into UniProt format, standardized, integrated, and subsequently de-duplicated in Excel to identify potential drug targets for further investigation.

### 2.7 Acquisition of disease molecules associated with degenerative disk disease

Focusing on the term “degenerative disk disease,” we conducted a search in the GeneCards database with a relevance score of 10 or higher for targets linked to this condition. We then consolidated the findings and eliminated any duplicates to curate a list of disease-related targets.

### 2.8 Mapping drug-disease molecular interactions and constructing a protein-protein interaction (PPI) network

To identify common targets between albumin and degenerative disk disease, we employed a Venn diagram tool. The overlapping targets were subsequently submitted to the String database, ensuring we selected “Homo sapiens” as the species and set the confidence score above 0.4 to establish PPI relationships. The resulting data was analyzed and visualized using Cytoscape version 3.7.1.

### 2.9 GO function and KEGG pathway enrichment analysis

The overlapping targets were evaluated using the DAVID database for Gene Ontology (GO) enrichment, while the corresponding pathways were identified through KEGG annotations. Only pathways exhibiting significance at *P* < 0.05 were selected for further clustering analysis. The top 10 GO terms and the 20 most noteworthy KEGG pathways were visualized on the microbiology platform^1^ adhering to the *P* < 0.05 significance threshold.

### 2.10 Molecular docking

Key targets were subjected to molecular docking with Chrysin. The three-dimensional structures of the target proteins were procured from the PubChem database. These structures were then refined using PyMOL 2.6.0 to eliminate original ligands, solvent molecules, and extraneous protein chains while also adding hydrogen atoms. Binding energies for the docked interactions were computed with Autodock Vina 1.1.2, with the docking runs set to 20 to ensure reliability and to examine the binding dynamics between Chrysin and the target. PyMOL was employed for a graphical representation of the molecular docking results.

### 2.11 Verification of the binding model using MDS

Molecular dynamics simulations were performed using GROMACS 2022.3. Small molecule preparation involved the following steps: molecular hydrogenation and restrained electrostatic potential (RESP) charge calculations were conducted using Gaussian 16, followed by assignment of the General Amber Force Field (GAFF) parameters through AmberTools22. These parameterized data were subsequently integrated into the system topology file. Simulations were conducted under constant temperature (300 K) and pressure (1 bar) conditions using the AMBER99SB-ILDN force field, with explicit solvation achieved through the TIP3P water model. Charge neutralization was accomplished by adding Na^+^ counterions. The system underwent energy minimization via the steepest descent algorithm, followed by sequential equilibration in NVT (constant particle number, volume, and temperature) and NPT (constant particle number, pressure, and temperature) ensembles. Each equilibration phase comprised 100,000 steps using a 0.1 ps coupling constant, totaling 100 ps per phase. Production dynamics were executed for 50 ns (5,000,000 steps at 2 fs/step). Post-simulation analysis utilized built-in GROMACS utilities to calculate root-mean-square deviation (RMSD), root-mean-square fluctuation (RMSF), radius of gyration, and binding free energy estimates via the MM/GBSA method, supplemented by free energy landscape analysis (33, 34).

### 2.12 Statistical analysis

All experimental procedures were performed in triplicate. Statistical analyses were conducted using SPSS 20.0 (IBM Corp.) with data presented as mean ± SD (*n* = 3). Intergroup comparisons were analyzed through Student’s *t*-test or one-way ANOVA, followed by Tukey’s *post-hoc* test when appropriate. Statistical significance threshold was established at *p* < 0.05.

## 3 Results

### 3.1 Target identification for Chrysin

Potential targets of chrysin were identified by searching the TCMSP, SuperPred, and Swiss Target Prediction databases. The resulting data were subjected to UniProt normalization, yielding 19,106, and 104 target molecules, respectively. Merging these datasets and removing any duplicates resulted in a final list of 199 potential target molecules.

### 3.2 Target prediction for degenerative disk disease

Targets associated with DDD were identified via a search of the GeneCards database. Following data merging and removal of duplicates, a total of 2,865 potential target molecules were obtained.

### 3.3 Drug-disease target mapping

**FIGURE 1:**
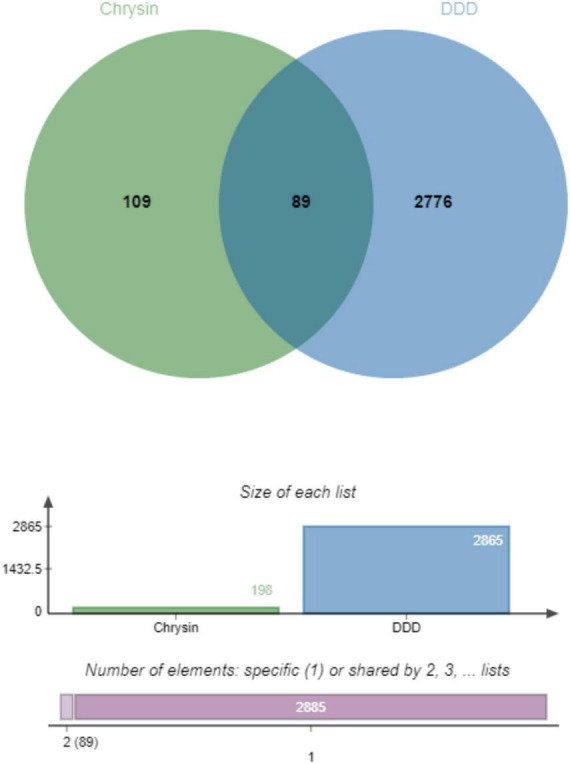
Identification of therapeutic targets of Chrysin against DDD, venn diagram of potential therapeutic targets, from the intersection of 109 drug targets and 2776 DDD-related targets, 89 therapeutic targets for Chrysin anti-DDD can be derived. DDD, intervertebral disk degeneration.

The component targets of chrysin and DDD-related targets were mapped using the Venn analysis tool, the intersection targets were extracted, and finally 89 drug-disease potential targets were obtained (Figure 1).

### 3.4 PPI network of target proteins

**FIGURE 2:**
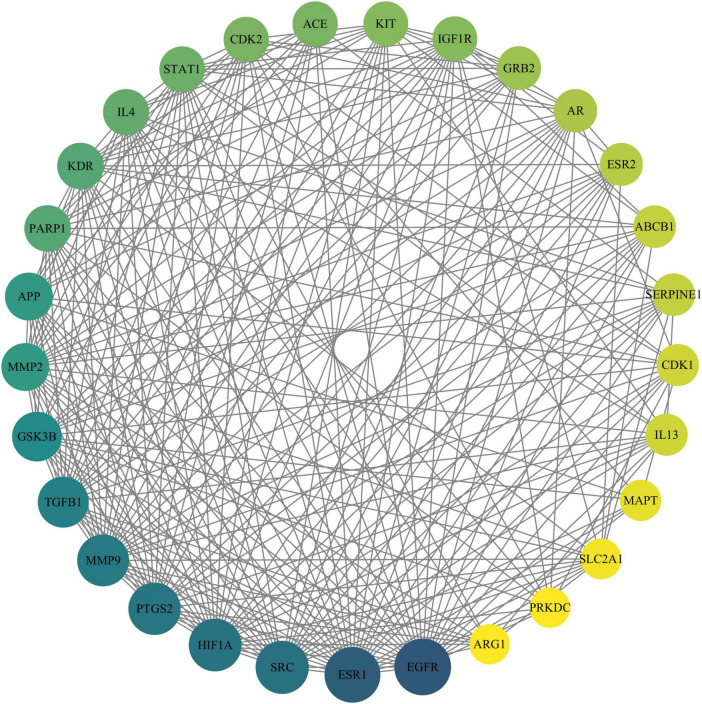
Protein–protein interaction network of Chrysin targets against DDD. The size of the circle reflects the degree of connectivity (a larger circle indicates a higher degree of connectivity). DDD, intervertebral disk degeneration.

The PPI network constructed using the STRING online database for 89 drug-disease action targets was analyzed, and the results showed that the network had 89 nodes and 716 edges, with an average node degree of 16.1 and PPI enrichment (*p* < 0.01). The PPI network constructed by String was imported into Cytoscape software, and five basic topological parameters, namely degree, betweenness centrality, closeness centrality, radiality, and stress, were analyzed using the CytoNCA built-in plugin, resulting in 29 target genes with top-ranking parameters. Furthermore, using a median value greater than five parameters as the screening criterion, 29 core targets were finally obtained, which are ABCB1, MMP2, KDR EGFR, KIT, GSK3B, PARP1, PTGS2, MMP9, SRC, ESR1, SLC2A1, HIF1A, IGF1R, ACE, TGFB1, SERPINE1, IL4, APP, MAPT, GRB2, ARG1, AR, ESR2, CDK1, STAT1, CDK2, PRKDC, and IL13 (Figure 2).

### 3.5 GO and KEGG enrichment analyses

**FIGURE 3:**
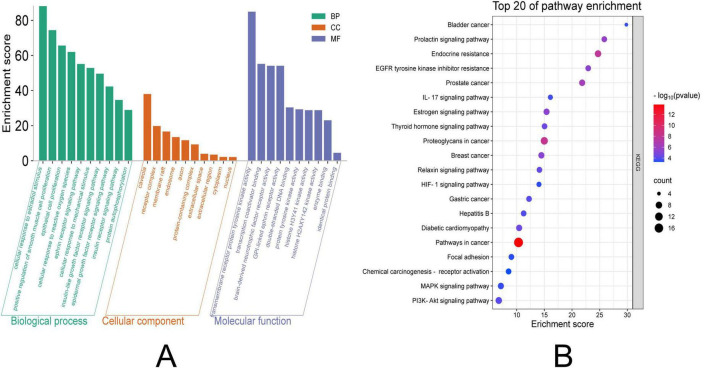
GO and KEGG enrichment analysis visualization. **(A)** The left panel displays GO terms categorized into three domains: MF, CC, and BP. For GO terms: The horizontal axis (Count) indicates the number of genes associated with each term; longer bars represent greater gene enrichment. **(B)** The right panel illustrates enriched KEGG pathways: The horizontal axis (Enrichment Factor) reflects the magnitude of enrichment. Dot size corresponds to the number of enriched genes, with larger dots indicating higher counts. Dot color intensity represents the statistical significance (–log10 adjusted *p*-value), where redder hues denote greater significance (*p* < 0.05).GO, Gene Ontology; KEGG, Kyoto Encyclopedia of Genes and Genomes. CC, Cellular Component. MF, Molecular Function. BP, Biological Process.

The intersecting target genes were analyzed through DAVID database for GO functional annotation and KEGG pathway enrichment (screened at *P* < 0.05) and visualized using Weishengxin online tools. GO Analysis: Biological Processes (BP): 251 terms enriched. Top 10 significant processes (*P*-value magnitude indicated enrichment strength): Negative regulation of gene expression, ephrin receptor signaling pathway, positive regulation of smooth muscle cell proliferation, response to xenobiotic stimulus. Cellular Components (CC): 38 terms enriched. Top 10 significant terms: Protein-containing complex, receptor complex, endosome, axon. Molecular Functions (MF): 80 terms enriched. Top 10 significant terms: Enzyme binding, transmembrane receptor protein tyrosine kinase activity, double-stranded DNA binding, identical protein binding (Figure 3A). KEGG Pathway Analysis: 72 pathways identified. Top 20 significant pathways (*P* < 0.05): Efferocytosis, central carbon metabolism in cancer, cell cycle, hepatitis C, JAK-STAT signaling pathway (Figure 3B). The complete results are presented in Supplementary Table 1.

### 3.6 Molecular docking and molecular dynamics simulations

**FIGURE 4:**
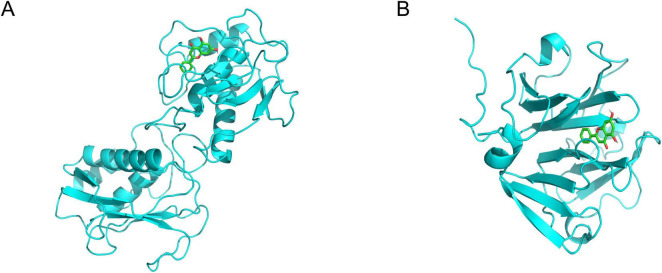
The 3-dimensional map of the binding sites between Chrysin and target proteins **(A)** MMP9, **(B)** MMP2. Chrysin is shown in yellow. Target proteins are displayed as cyan. The places where Chrysin and the target proteins are connected represent specific docking sites between Chrysin and target proteins.

The binding affinity of Chrysin with target proteins was predicted using the SwissDock web server. The docking models were based on the lowest recorded binding energies (Delta G). We selected MMP9 and MMP2 (Figures 4A, B), which we had previously validated through experimentation, for molecular docking with Chrysin. The results indicated that the minimum binding energies for MMP9 and MMP2 with RES were –8.2 and –8.4 kcal/mol, respectively, both below –5.0 kcal/mol, suggesting a strong binding activity. 2D interaction diagrams (hydrogen bonding, hydrophobic/π–π contact) have been added to Supplementary Figure 1 to verify the binding of MMP9 active sites.

**FIGURE 5:**
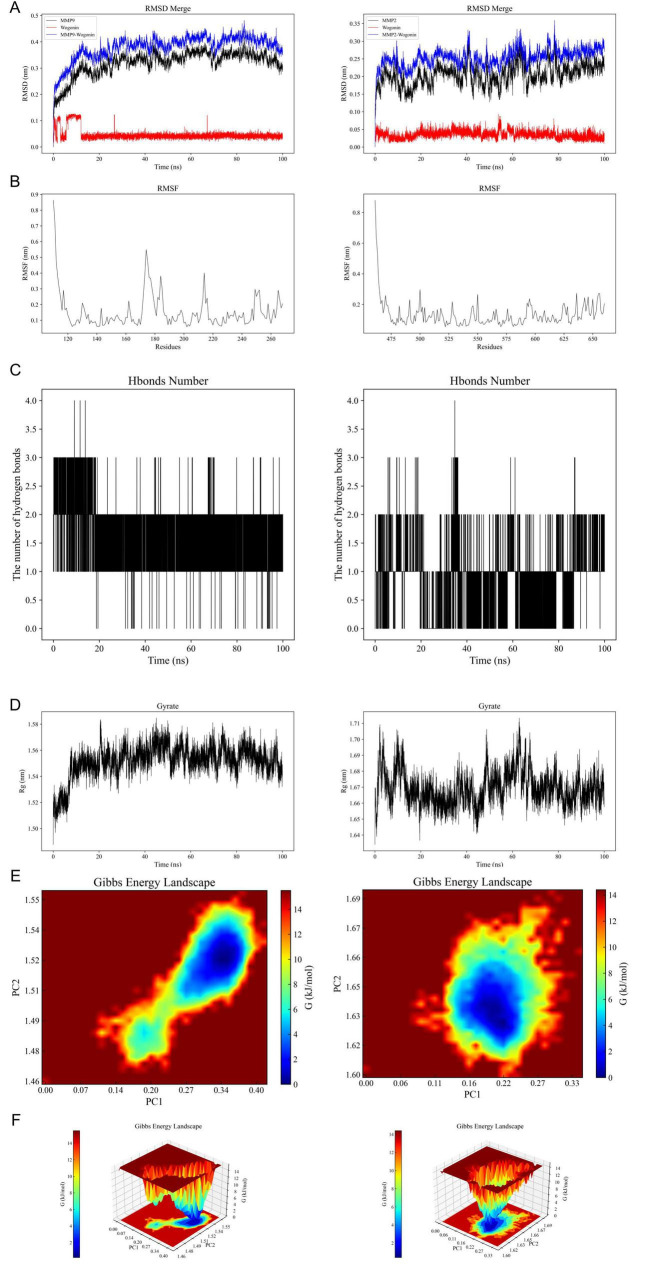
Results of molecular dynamics simulations (MDS) involving Chrysin and target proteins. **(A)** The RMSD values for Chrysin-MMP9 complex (left) and Chrysin-MMP2 complex (right). **(B)** Variations in protein flexibility throughout the Chrysin simulation. MMP9 (left), MMP2 (right). **(C)** Dynamics of hydrogen bonding as observed in the molecular dynamics simulations. MMP9 (left), MMP2 (right). **(D)** Rg rate curve of the protein-Chrysin complex. MMP9 (left), MMP2 (right). **(E,F)** Two-dimensional and three-dimensional mappings of the free energy landscape. MMP9 (left), MMP2 (right). RMSD, root-mean-square deviation; RMSF, root-mean-square fluctuation; ΔMMGBSA, Δ molecular mechanics-generalized-Born surface area; Rg, radius of gyration; PC, principal component.

All-atom molecular dynamics simulations (MDS) were conducted for 100 ns using GROMACS to investigate protein flexibility and Chrysin-protein complex stability. Post-simulation trajectory analysis with built-in tools quantified six parameters: root-mean-square deviation (RMSD), root-mean-square fluctuation (RMSF), hydrogen bond occupancy, MMGBSA free energy (ΔMMGBSA), radius of gyration (Ryrate), and free-energy topography. The RMSD trajectories exhibited minimal fluctuations (< 2.5 Å) across all simulated complexes, indicating structural stability (Figure 5A). Residue-specific flexibility patterns were revealed through RMSF analysis, with peak variations localized in loop regions (Figure 5B). Persistent hydrogen bonding (> 75% occupancy) was observed in the MMP9-Chrysin complex (Figure 5C). Ryrate analysis demonstrated stable compactness (ΔRyrate < 1.2 Å) with consistent molecular packing throughout simulations (Figure 5D). Principal component analysis of RMSD and Ryrate values identified two dominant motion modes. Projection of these modes onto Gibbs free energy landscapes showed distinct low-energy basins (ΔG < –10 kcal/mol), confirming thermodynamic stability (Figures 5E, F). Collectively, these analyses establish favorable kinetic profiles for Chrysin complexes.

### 3.7 Effect of chrysin on H_2_O_2_-induced senescence model of NPCs

**FIGURE 6:**
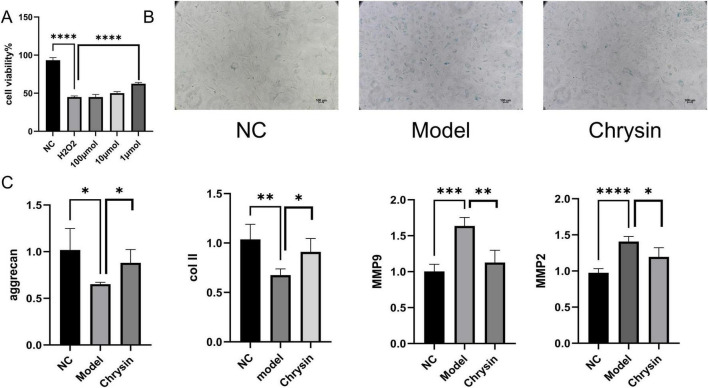
Chrysin-containing serum ameliorates senescence of NPCs under H_2_O_2_ intervention. Effects of different doses of Chrysin on NPCs viability **(A)**; Representative images of SA-β-gal staining of NPCs **(B)**; COL II, aggrecan, MMP9, and MMP2 mRNA expression levels **(C)** NPCs, nucleus pulposus cell. Each experiment was conducted thrice, and the data are shown as the mean ± SD. *p < 0.05, **p < 0.01. ***p < 0.001, ****p < 0.0001. NC: control groups, Model: H2O2-induced NPCs senescence models, chrysin: the addition of chrysin in the H2O2-induced NPCsl senescence model.

To assess the protective role of serum enriched with Chrysin on H_2_O_2_-induced senescent myeloid cells, we conducted a study evaluating how Chrysin influences the viability of H_2_O_2_-induced senescent myeloid cells using the CCK8 assay. Our results show that Chrysin containing 1 μmol concentration exhibits significant protection against H_2_O_2_-induced NPCs senescence models. Consequently, this particular concentration was selected for all future experiments, as illustrated in Figure 6A. The SA-β-gal staining indicated that the proportion of blue-stained cells in the normal group was notably lower compared to the model group, while the model group exhibited a higher percentage of blue-stained cells than the Chrysin group, as demonstrated in Figure 6B. Furthermore, results from RT-qPCR showed that the expression of MMP9 and MMP2 mRNA levels in the NPCs of the normal group were reduced relative to those in the model group. Additionally, the NPCs of the Chrysin group also displayed lower expression levels of MMP9 and MMP2 compared to the model group. Conversely, the expression levels of COL II and ACAN mRNA in the NPCs of the normal group were elevated in comparison to the model group, while the NPCs of the Chrysin group also showed increased levels of COL II and ACAN mRNA compared to the model group (Figure 6C).

## 4 Discussion

Low back pain (LBP), largely driven by DDD, remains a global health burden with limited therapeutic options targeting its underlying pathology (1). Our study provides the first evidence that chrysin, a natural flavonoid, alleviates DDD progression by dual inhibition of MMPs and NPCs senescence, bridging network pharmacology predictions with functional validation.

The key finding—chrysin’s suppression of MMP2/9 expression and collagen II/aggrecan restoration—aligns with prior reports on flavonoids (e.g., hesperidin, naringin) targeting ECM dysregulation (27, 28). However, unlike other polyphenols limited to single pathways, chrysin uniquely disrupts the senescence-MMPs feedback loop. Senescent NPCs secrete SASP factors (IL-6, HMGB1) that amplify MMP activity, perpetuating ECM degradation (4). By reducing SA-β-gal positivity and SASP markers (p16INK4a), chrysin not only inhibits MMP2/9 but also breaks this vicious cycle, offering a multi-target advantage over conventional MMP inhibitors prone to off-target effects (5). Molecular docking further corroborates its strong binding affinities to MMP2 (–8.4 kcal/mol) and MMP9 (–8.2 kcal/mol), surpassing reported values for synthetic inhibitors like prinomastat (6). This specificity may stem from chrysin’s interaction with conserved catalytic zinc-binding sites, a hypothesis warranting crystallographic validation.

Our network pharmacology analysis identified TGFB1, NF-κB, and JAK-STAT as central pathways linking chrysin’s anti-senescence and anti-MMP effects. TGFB1, a dual regulator of ECM synthesis and fibrosis (7), was downregulated by chrysin, suggesting its role in balancing anabolism/catabolism. Meanwhile, chrysin’s suppression of NF-κB aligns with its known inhibition of ROS/IKKβ signaling (9), potentially blocking MMP transcription. Notably, the Nrf2/HO-1 axis—a master regulator of oxidative stress—was enriched in KEGG results but requires experimental validation to clarify its crosstalk with senescence.

Despite these advances, limitations exist. First, our H_2_O_2_-induced senescence model mimics oxidative stress but overlooks mechanical loading essential to DDD pathogenesis (10). Second, while chrysin’s efficacy at 1 μM was optimal *in vitro*, pharmacokinetic studies are needed to determine achievable concentrations in the hypoxic disk niche. Third, the exclusion of apoptosis modulators (e.g., BCL2 inhibitors) may underestimate chrysin’s broader anti-degenerative effects. Future work should integrate dynamic compression systems and *in vivo* models to evaluate chrysin’s spatial-temporal efficacy. Clinically, our findings position chrysin as a candidate for localized disk therapy. Its poor oral bioavailability (23) necessitates innovative delivery systems (e.g., hydrogel microspheres) to sustain intra-disk concentrations.

## 5 Conclusion

Chrysin alleviates intervertebral disk degeneration (DDD) by dual targeting of MMP-mediated ECM degradation and NPC senescence. Network pharmacology identified 89 key targets (e.g., MMP2/9, TGF-β), validated experimentally through reduced SA-β-gal activity and MMP2/9 suppression, alongside restored collagen II/aggrecan. Molecular docking revealed high-affinity binding to MMP2/9 (–8.4/–8.2 kcal/mol). Molecular dynamics simulations demonstrated that the Chrysin-MMP-9 and Chrysin-MMP-2 complexes exhibited favorable dynamic properties. These findings highlight chrysin’s multi-target advantage over single-pathway therapies.

## Data Availability

The datasets presented in this study can be found in online repositories. The names of the repository/repositories and accession number(s) can be found in the article/Supplementary material.

## Footnotes

1
https://www.bioinformatics.com.cn/

## Supplementary material

Supplementary Table 1 GO and KEGG enrichment analyses.

Supplementary Figure 1 2D interaction diagrams (hydrogen bonding, hydrophobic/π–π contact).
