# Supplementary material for: Chrysin attenuates intervertebral disk degeneration via dual inhibition of matrix metalloproteinases and senescence: integrated network pharmacology, molecular docking, and experimental validation
Source: Front Med (Lausanne). 2025 May 12;12:1593317. doi: 10.3389/fmed.2025.1593317 (PMC12104227; doi:10.3389/fmed.2025.1593317)
Supplement: Supplementary Figure 1 — 2D interaction diagrams (hydrogen bonding, hydrophobic/π–π contact). [file Supplementary_file_1.docx]

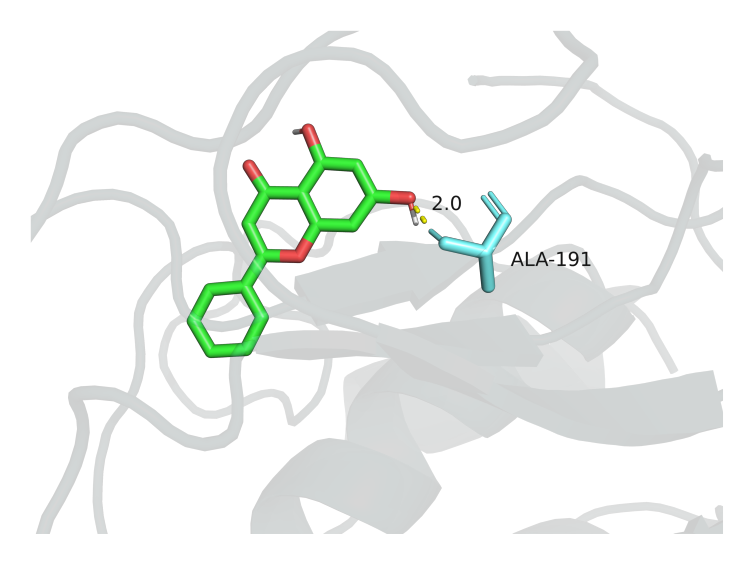


2D Ligand Interaction Diagram of Chrysin Binding to the MMP-9 Active Site (Hydrogen Bonds, Hydrophobic/π-π Contacts)
